# Traumatic perinatal events and educational needs of labor and delivery room nurses in Korea: a cross-sectional survey

**DOI:** 10.4069/whn.2024.03.10

**Published:** 2024-03-29

**Authors:** Nagyeong Lee, Gunjeong Lee

**Affiliations:** College of Nursing, Ewha Womans University, Seoul, Korea

**Keywords:** Delivery rooms, Education, Nurse, Perinatal care

## Abstract

**Purpose:**

The present study investigated experiences of traumatic perinatal events, the provision of related education, and educational needs of nurses working in the labor and delivery room (LDR).

**Methods:**

Nurses working in the LDRs of six institutions and two nurse portal sites were invited to participate in the survey, delivered on paper or online. The data were collected from October 1 to November 25, 2022. Data from 129 nurses were analyzed using frequency, the chi-square test, the Fisher exact test, the t-test, and analysis of variance.

**Results:**

Virtually all participants (98.6%) reported having experienced at least one traumatic perinatal event (dystocia, postpartum hemorrhage, neonatal congenital anomalies, severe maternal or neonatal injury, stillbirth, and maternal or neonatal death) while working in the LDR. The most shocking traumatic perinatal event experienced was the maternal or neonatal death (40.3%), but 24.8% of participants did not recall ever receiving education on the topic. About 63% of participants experienced traumatic perinatal events within a year of working in the LDR. The average score for education needs regarding traumatic perinatal events was 3.67±0.37 out of 4, and participants preferred simulation education as the most effective educational method.

**Conclusion:**

Since most of the participants had experienced various traumatic perinatal events in the early stages of working in the LDR and expressed a high level of need for education on traumatic perinatal events, it is necessary to provide more effective stimulation education programs in the early period of work in the LDR.

## Introduction

Nurses working in labor and delivery units frequently experience various traumatic events during their shifts, involving maternal or neonatal death, stillbirth, severe maternal or neonatal injury, neonatal congenital anomalies, postpartum hemorrhage, and dystocia [1-3]. In South Korea, as the average age of first-time mothers has increased, the incidence of high-risk pregnancies has also risen [4,5], leading to more traumatic perinatal events [6]. According to international studies, over 70% of labor and delivery nurses have experienced such events [2]. As high-risk deliveries increase, nurses are more likely to experience or witness traumatic perinatal events, potentially leading to adverse psychological reactions like posttraumatic stress, burnout, increased turnover intention, and compromised nursing care quality [3,5]. Therefore, educating nurses on appropriate responses and coping strategies for emotional distress after such experiences is crucial [7].

To respond appropriately, nurses must know about traumatic perinatal events and have excellent clinical performance skills [8]. While nursing education enhances clinical competence and confidence in practice [9], previous studies have reported lower labor and delivery nursing performance among nurses than midwives, as nurses are often assigned without systematic training [10,11]. Limited educational opportunities exist in smaller hospitals and clinics [12]. Additionally, due to declining birth rates, nursing students have fewer opportunities to witness actual deliveries during clinical practicum [13,14], leading to newly graduated nurses potentially needing more clinical performance skills.

A previous study confirmed a high demand for education on traumatic perinatal events among nurses in high-risk maternal-neonatal integrated care centers [10]. Nurses working in smaller institutions were found to have limited opportunities for professional development [10,15]. However, research on labor and delivery nurses’ actual experiences with traumatic perinatal events and the provision of related education in clinical practice still needs to be improved. It is necessary to explore the current state of traumatic perinatal events, the provision of related education, educational needs, and effective educational methods to provide appropriate clinical performance education.

The purpose of this study is to investigate labor and delivery nurses’ experiences with various traumatic perinatal events, educational status, and educational needs to develop appropriate training programs. The specific objectives are as follows:

1. To identify labor and delivery nurses’ experiences with traumatic perinatal events, the provision of related education, and educational needs regarding traumatic perinatal events.

2. To examine differences in the experiences of traumatic perinatal events, the provision of related education, and educational needs according to the general characteristics of labor and delivery nurses.

## Methods

Ethics statement: This study was approved by the Institutional Review Board of Ewha Womans University (No. ewha-202209-0018-01). Informed consent was obtained from the participants.

### Study design

This study employed a descriptive correlational survey design to analyze labor and delivery nurses’ experiences with traumatic events, educational status, and educational needs. This study adhered to the STROBE (STrengthening the Reporting of OBservational studies in Epidemiology) reporting guidelines (https://www.strobe-statement.org/).

### Participants

The participants were labor and delivery nurses recruited through convenience sampling from across Korea who understood the purpose and methods of the study and voluntarily agreed to participate. Nurses on leave were excluded from the study. Based on a previous study on educational needs [16] and using G-power 3.1.9.7 with a significance level of .05, an effect size of .30, and a power of .80, the minimum sample size required for analysis of variance with four groups (the maximum number of groups for work experience) was 128. Considering a 5% to 10% dropout rate [16,17], 136 participants were initially recruited. After excluding seven inappropriate or insincere responses, data from 129 participants (60 offline and 69 online) were analyzed.

### Measurement

#### Traumatic perinatal events

Traumatic perinatal events were measured by assessing the experience of six events (dystocia, postpartum hemorrhage, neonatal congenital anomalies, severe maternal or neonatal injury, stillbirth, and maternal or neonatal death) selected from the lists provided by Sheen et al. [2] and Çankaya and Dikmen [3]. It included seven traumatic perinatal events identified by Çankaya and Dikmen [3] (dystocia, neonatal congenital anomalies, severe maternal or neonatal injury, stillbirth, maternal or neonatal death, maltreatment of attending medical staff treatment during delivery, and disregarding maternal requests during delivery) and the five characteristics of traumatic perinatal events described by Sheen et al. [2] (stillbirth, postpartum hemorrhage, severe maternal or neonatal injury, neonatal death due to dystocia, and permanent or temporary complications due to delivery). The four events common to both studies (dystocia, severe maternal or neonatal injury, stillbirth, and maternal or neonatal death) were initially selected as traumatic perinatal events. The two events related to medical staff maltreatment and disregarding maternal requests from Çankaya and Dikmen [3] were excluded as they were not direct traumatic events. In contrast, the “permanent or temporary complications due to delivery” event from Sheen et al. [2] was excluded because it is challenging to confirm quickly. Additionally, “neonatal congenital anomalies” [3,17] and “postpartum hemorrhage” [2,8,17], which are included in various studies on traumatic perinatal events, were added to the final selection of six events. The participants responded dichotomously (“yes” or “no”) to indicate their experience with each event. The Cronbach’s α for this measure was .66.

#### Educational status on traumatic perinatal events

The educational status was assessed by asking participants whether they had received education on clinical management approaches for each of the six traumatic perinatal events while working in the labor and delivery unit. The response options were “received education, and it was helpful for practice,” “received education, but it was not helpful for practice,” and “did not receive education.” The Cronbach’s α for this measure was .88.

#### Educational needs for traumatic perinatal events

The educational needs for traumatic perinatal events were measured by assessing the participants’ perceived need for education on the latest medical knowledge-based clinical management approaches for each of the six traumatic perinatal events. The responses were rated on a 4-point Likert scale ranging from 1 (“not needed at all”) to 4 (“highly needed”), with higher scores indicating more significant educational needs. The Cronbach’s α for this measure was .81. Additionally, the participants were asked about the most effective educational method for addressing traumatic perinatal events in practice.

#### General characteristics

The general characteristics included sex, age, highest educational level, midwifery license status, current workplace, clinical work experience, and labor and delivery unit work experience.

### Data collection

Data were collected from October 1 to November 25, 2022, through offline and online surveys of labor and delivery nurses who voluntarily agreed to participate in the study. For the offline survey, nurses from six accessible tertiary hospitals in the metropolitan area were conveniently recruited. Before conducting the survey, the researchers explained the study purpose to each hospital’s nursing department and head nurses and obtained approval for data collection. The participants were then informed about the study purpose and methods, and only those who voluntarily consented to participate were included in the offline survey. The online survey was conducted after the offline survey by posting recruitment notices on nursing community portals (Nurscape and NursesStory). Participants could voluntarily access the online survey through the provided URL or QR code. The online survey was only accessible to nurses who had not participated in the offline survey to prevent duplicate participation. The general characteristics of the offline and online participants were analyzed, and no significant differences were found.

### Data analysis

The collected data were analyzed using IBM SPSS version 26.0 (IBM Corp., Armonk, NY, USA) as follows:

1. The participants’ general characteristics were analyzed using frequencies, percentages, means, and standard deviations.

2. The participants’ experiences with traumatic perinatal events, educational status, educational needs, and preferred effective educational methods were analyzed using frequencies, means, and standard deviations.

3. Differences in the experiences of traumatic perinatal events and educational status according to the participants’ general characteristics were analyzed using the chi-square tests and Fisher exact tests.

4. Differences in educational needs for traumatic perinatal events according to the participants’ general characteristics were analyzed using independent t-tests and one-way analysis of variance, followed by Scheffé post-hoc tests.

## Results

### Participants

Most participants were female nurses in the 20-29 years (48%) and 30-39 years (41.9%) age groups. Thirty-five (27.1%) had a midwife license (Table 1).

### Experiences with traumatic perinatal events

The most frequently experienced traumatic perinatal event was postpartum hemorrhage (93.8%), followed by dystocia (77.5%), stillbirth (76.0%), neonatal congenital anomalies (72.1%), severe maternal or neonatal injury (50.4%), and maternal or neonatal death (49.6%) (Table 2).

Overall, 98.4% of the participants had experienced at least one traumatic perinatal event, with 70.5% experiencing four or more of the six types of events. On average, the participants had experienced 4.19 traumatic perinatal event domains. The most shocking event was maternal or neonatal death (40.3%), and the majority (68.2%) had experienced the most shocking event within the past year, followed by within 5 years (20.9%), more than 5 years (7.0%), and more than 10 years (2.3%) (Table 2).

### Educational status on traumatic perinatal events

The highest proportion of participants who had not received education was for maternal or neonatal death (24.8%), followed by neonatal congenital anomalies (22.5%) and severe maternal or neonatal injury (20.2%). Among those who had received education, approximately 20% reported that the education was not helpful for practice, with the highest proportions for neonatal congenital anomalies, dystocia, stillbirth, and maternal or neonatal death (Table 2).

### Educational needs for traumatic perinatal events

The mean educational need for traumatic perinatal events was high at 3.67 (out of 4)±0.37, with all six events scoring above 3. The highest educational need was for postpartum hemorrhage (3.82±0.44), followed by maternal or neonatal death (3.74±0.46), stillbirth (3.71±0.50), severe maternal or neonatal injury (3.69±0.48), neonatal congenital anomalies (3.62±0.51), and dystocia (3.46±0.70) (Table 3).

Additionally, 62% of the participants indicated that simulation training would be the most effective educational method for addressing traumatic perinatal events, followed by lectures (16.3%), case studies (13.2%), and video viewing (8.5%), showing a strong preference for simulation training (Table 2).

### Differences in experiences of traumatic perinatal events based on general characteristics

Analyses of differences based on age revealed that participants in their 40s or older had experienced more neonatal congenital anomalies (*p*=.021) than younger participants. Educational level was associated with differences in the experience of maternal or neonatal death (*p*=.005), with participants who graduated from 3-year college programs reporting significantly lower frequencies of experiencing this event. Regarding midwifery license status, licensed midwives had higher frequencies of experiencing neonatal congenital anomalies (*χ*^2^=4.43, *p*=.035) and maternal or neonatal death (*χ*^2^=4.98, *p*=.026). The hospital type was associated with differences in the experiences of neonatal congenital anomalies (*χ*^2^=7.66, *p*=.022) and stillbirth (*χ*^2^=6.83, *p*=.033), with participants working at tertiary hospitals reporting significantly higher frequencies. Labor and delivery unit work experience was related to differences in the experiences of dystocia (*χ*^2^=8.77, *p*=.033), postpartum hemorrhage (*p*<.001), neonatal congenital anomalies (*p*<.001), stillbirth (*p*<.001), and maternal or neonatal death (*p*=.002), with more extended work experience being associated with higher frequencies of experiencing these events. Similarly, longer overall clinical work experience was associated with higher frequencies of experiencing postpartum hemorrhage (*p*<.001), neonatal congenital anomalies (*p*<.001), stillbirth (*p*=.018), and maternal or neonatal death (*p*=.034) (Table 4).

### Differences in traumatic perinatal events education based on general characteristics

In terms of age, a difference was found in maternal and neonatal death (*χ*^2^=7.74, *p*=.021), with those in their 40s or older having a higher rate of receiving education on traumatic events. Depending on midwifery license status, differences were observed in dystocia (*p*=.011), postpartum hemorrhage (*p*=.036), neonatal malformation (*p*=.004), severe maternal or neonatal injury (*χ*^2^=6.22, *p*=.013), stillbirth (*p*=.036), and maternal or neonatal death (*χ*^2^=6.78, *p*=.009), with higher education rates among those with a midwifery license. Based on labor unit work experience, differences were noted in dystocia (*p*=.009), neonatal malformation (*p*=.011), severe maternal or neonatal injury (*p*=.002), and maternal or neonatal death (*p*=.011), with nurses working for less than a year having lower education rates. No differences in traumatic perinatal event education were observed based on education level, current hospital, or total clinical work experience (Table 5).

### Differences in educational needs for traumatic perinatal events based on general characteristics

Differences were found in dystocia (F=10.56, *p*<.001), postpartum hemorrhage (F=6.41, *p*=.002), and stillbirth (F=5.52, *p*=.005) based on education level. Post-hoc analysis showed that those who graduated from a 4-year university had higher educational needs than those from a 3-year college. Participants with a midwifery license had higher educational needs for neonatal malformation (t=4.65, *p*<.001), severe maternal or neonatal injury (t=2.88, *p*=.005), stillbirth (t=3.15, *p*=.002), and maternal or neonatal death (t=2.11, *p*=.037). Additionally, differences were observed in maternal or neonatal death (F=3.84, *p*=.024) based on the current hospital. Post-hoc analysis revealed that nurses working at advanced general hospitals had higher educational needs for maternal or neonatal death than those at general hospitals. Educational needs for maternal or neonatal death differed based on labor unit work experience (F=3.31, *p*=.022). Post-hoc testing showed that labor unit nurses with 3 or more years of experience had higher educational needs for maternal or neonatal death than those with 1-3 years of experience. Differences were found in dystocia (F=6.02, *p*=.001) and maternal or neonatal death (F=3.82, *p*=.012) based on total clinical work experience. Post-hoc analysis indicated that labor unit nurses with 5 or more years of experience had higher educational needs than those with 3 to 5 years of experience (Table 3).

## Discussion

This study aimed to provide essential data for developing appropriate educational programs by identifying traumatic perinatal events experienced by labor unit nurses, their education status, and their educational needs. In this study, 98.4% of labor unit nurses experienced one or more traumatic perinatal events. The prevalence of such experiences varies across studies based on the definition and range of events considered. For instance, Wahlberg et al. [18] reported that 71% of Swedish labor unit nurses experienced maternal or neonatal death or severe maternal or neonatal injury, defined as traumatic perinatal events. Among the participants, 50.4% experienced severe maternal or neonatal injury, 49.6% experienced maternal or neonatal death. Of those who experienced both events, the percentage was 69.8%, which was similar to this study. Also, in a Chinese study by Qu et al. [19], where dystocia, postpartum hemorrhage, maternal and neonatal death, and severe maternal or neonatal injury were considered traumatic perinatal events, 98.1% of labor unit nurses experienced them at least once during their work. The present study found comparable percentages for these events.

Notably, half of the respondents had 3 years or less of labor unit experience, and even nurses with less than 1 year reported encountering traumatic perinatal events. However, these nurses had lower education rates for events like dystocia, neonatal malformation, severe maternal or neonatal injury, and maternal or neonatal death while scoring high educational needs (≥3 out of 4) for such events, indicating an urgent need for practical training before or early in their labor unit assignment.

Differences in neonatal malformation experience were observed based on age, labor unit experience, midwifery license status, and working at an advanced general hospital. This is likely due to increased exposure to difficult deliveries and higher rates of diagnosis at advanced facilities. About 4.3% of newborns were reported as severe neonatal congenital anomalies in tertiary hospital [20].

Maternal or neonatal death was the most traumatic perinatal event, with 24.8% of participants lacking education on this topic, the highest among events. Given the potential for such events to occur and the high educational need (3.74 out of 4), education on maternal or neonatal death appears urgently required, particularly for experienced nurses with 5 or more years of clinical experience, who were more likely to care for critically ill patients.

Previous study [21] found simulation-based education covering the nursing process, emotional support, psychological fears, and stress management strategies for maternal or neonatal death care effective in clinical practice. Therefore, research on the emotional and psychological challenges labor unit nurses face during such care, and the development of educational programs based on these findings is necessary. Additionally, as nurses who experience or witness traumatic perinatal events are at higher risk of burnout, posttraumatic stress, and increased turnover intention [11,22], research on psychological programs and healing/rehabilitation programs to prevent turnover is needed.

Participants with a midwifery license received more education on all traumatic perinatal events, likely due to their extended labor unit experience and more specific perinatal education than undergraduate nursing programs. While the Korean Nurses Association and Korean Midwives Association offer continuing education on related topics [23,24], additional content covering a more comprehensive range of traumatic perinatal events is needed, incorporating effective education methods reported in international studies [21], such as appropriate use of medical equipment, time management, role distribution in emergencies, and proper communication methods.

Educational needs for traumatic perinatal events scored high (≥3 out of 4) for all items, consistent with findings from Kim et al. [11]. Higher educational needs for dystocia, postpartum hemorrhage, and stillbirth were observed among 4-year university graduates compared to 3-year college graduates, potentially due to differences in patient acuity levels at their workplaces. Educational needs of maternal or neonatal death were higher among nurses working at a tertiary hospital, who were more likely to experience maternal or neonatal death than those working at a general hospital.

Respondents expected simulation-based education to be the most effective. Previous studies [25-27] reported improvements in knowledge, clinical practice skills, and nursing performance confidence related to traumatic perinatal events like postpartum hemorrhage after simulation-based education for practicing nurses. Simulation-based childbirth nursing education also positively affected nursing performance among South Korean nursing students [28,29]. Therefore, developing scenario-based simulation education programs for labor unit nurses, mainly focusing on maternal or neonatal death, postpartum hemorrhage, and stillbirth, is necessary. While the Korean Nurses Association provides simulation-based training for labor unit nurses with less than 1 year of experience, no simulation education specifically addressing traumatic perinatal events is available [30]. Hence, increasing training frequency, expanding the target audience to include all labor unit nurses, and prioritizing developing effective simulation-based educational programs are crucial.

This study has limitations in terms of the potential for biased sampling. Participants may have been more likely to have online access and offline data collection was limited to the metropolitan area. Therefore, generalization is limited, and further research with larger samples is necessary. Additionally, as the study investigated education received during labor unit work, recall bias may have occurred among nurses with longer labor unit experience, suggesting the need for future studies to focus on a specific period.

In conclusion, most labor unit nurses experience one or more traumatic perinatal events and have high educational needs regarding such events. In particular, practical training is urgently needed for appropriate response to various traumatic perinatal events before or early in labor unit assignments, as even nurses with less than 1 year of labor unit experience have already encountered these events yet had lower education rates, while their educational needs were high.

## Figures and Tables

**Table 1.** General characteristics of participants (N=129)

Variable	Categories	n (%)
Sex	Female	126 (97.7)
	Male	3 (2.3)
Age (year)	20–29	62 (48.0)
	30–39	54 (41.9)
	≥40	13 (10.1)
Education level	3-year college	21 (16.3)
	4-year university	103 (79.8)
	Graduate school	5 (3.9)
Midwife license	Yes	35 (27.1)
	No	94 (72.9)
Type of hospital	Primary	12 (9.3)
	Secondary	89 (69.0)
	Tertiary general	28 (21.7)
Employment duration in the labor and delivery room (year)	<1	9 (7.0)
	1–2	52 (40.3)
	3–4	37 (28.7)
	5–9	20 (15.5)
	≥10	11 (8.5)
Total clinical experience (year)	<1	4 (3.1)
	1–2	14 (10.9)
	3–4	46 (35.7)
	5–9	44 (34.1)
	≥10	21 (16.2)

**Table 2.** Experience of traumatic perinatal events and experience of education on traumatic perinatal events (N=129)

Variable	Categories	n (%)
***Experience of traumatic perinatal events***		
Postpartum hemorrhage	Yes	121 (93.8)
	No	8 (6.2)
Dystocia	Yes	100 (77.5)
	No	29 (22.5)
Stillbirth	Yes	98 (76.0)
	No	31 (24.0)
Neonatal congenital anomalies	Yes	93 (72.1)
	No	36 (27.9)
Severe maternal or neonatal injury	Yes	65 (50.4)
	No	64 (49.6)
Maternal or neonatal death	Yes	64 (49.6)
	No	65 (50.4)
The most traumatic perinatal event		
Maternal or neonatal death		52 (40.3)
Postpartum hemorrhage		24 (18.6)
Stillbirth		21 (16.3)
Neonatal congenital anomalies		11 (8.6)
Dystocia		10 (7.7)
Severe maternal or neonatal injury		7 (5.4)
Others		4 (3.1)
Timing of the occurrence of the most traumatic perinatal event (year)	<1	88 (68.2)
	1–4	27 (20.9)
	5–9	9 (7.0)
	≥10	3 (2.3)
Average number of traumatic perinatal events	Mean±SD	4.19±1.58
Types of experiences with traumatic perinatal events	None	2 (1.5)
	1 type	4 (3.1)
	2 types	20 (15.5)
	3 types	12 (9.3)
	4 types	28 (21.7)
	5 types	29 (22.5)
	6 types	34 (26.4)
***Experience of education on traumatic perinatal events***		
Dystocia	Received education and deemed it helpful	97 (75.1)
	Received education and deemed it unhelpful	18 (14.0)
	No education offered	14 (10.9)
Postpartum hemorrhage	Received education and deemed it helpful	105 (81.4)
	Received education and deemed it unhelpful	12 (9.3)
	No education offered	12 (9.3)
Neonatal congenital anomalies	Received education and deemed it helpful	81 (62.8)
	Received education and deemed it unhelpful	19 (14.7)
	No education offered	29 (22.5)
Severe maternal or neonatal injury	Received education and deemed it helpful	90 (69.8)
	Received education and deemed it unhelpful	13 (10.0)
	No education offered	26 (20.2)
Stillbirth	Received education and deemed it helpful	102 (79.1)
	Received education and deemed it unhelpful	15 (11.6)
	No education offered	12 (9.3)
Maternal or neonatal death	Received education and deemed it helpful	82 (63.6)
	Received education and deemed it unhelpful	15 (11.6)
	No education offered	32 (24.8)
Learning method that would be most helpful	Simulation	80 (62.0)
	Lecture	21 (16.3)
	Case study	17 (13.2)
	Video	11 (8.5)

**Table 3.** Differences in educational needs for traumatic perinatal events according to general characteristics (N=129)

Variable	Categories	Dystocia		Postpartum hemorrhage		Neonatal congenital anomalies		Severe maternal or neonatal injury		Stillbirth		Maternal or neonatal death	
		Mean±SD	t or F (*p*)	Mean ±SD	t or F (*p*)	Mean ±SD	t or F (*p*)	Mean ±SD	t or F (*p*)	Mean ±SD	t or F (*p*)	Mean ±SD	t or F (*p*)
Total		3.46±0.70		3.82±0.44		3.62±0.51		3.69±0.48		3.71±0.50		3.74±0.46	
Age (year)	20–29	3.45±0.69	0.09 (.909)	3.84±0.41	0.65 (.522)	3.53±0.53	1.88 (.157)	3.68±0.47	1.77 (.174)	3.66±0.54	0.54 (.583)	3.71±0.49	0.47 (.624)
	30–39	3.44±0.76		3.78±0.50		3.69±0.50		3.65±0.52		3.76±0.47		3.74±0.44	
	≥40	3.54±0.51		3.92±0.27		3.77±0.43		3.92±0.27		3.69±0.48		3.85±0.37	
Education level	3-year college^a^	2.86±1.15	10.56 (<.001)	3.52±0.60	6.41 (.002)	3.52±0.62	0.68 (.507)	3.52±0.60	1.57 (.210)	3.38±0.66	5.52 (.005)	3.57±0.50	2.32 (.102)
	4-year university^b^	3.58±0.51	(a<b)^†^	3.87±0.38	(a<b)^†^	3.63±0.50		3.72±0.45		3.77±0.44	(a<b)^†^	3.76±0.45	
	Graduate school^c^	3.40±0.54		4.00±0.00		3.80±0.44		3.80±0.45		3.80±0.44		4.00±0.00	
Midwife license	Yes	3.60±0.49	1.69 (.093)	3.83±0.38	0.11 (.900)	3.89±0.32	4.65 (<.001)	3.86±0.35	2.88 (.005)	3.89±0.32	3.15 (.002)	3.86±0.35	2.11 (.037)
	No	3.40±0.76		3.82±0.46		3.52±0.54		3.63±0.50		3.64±0.54		3.69±.048	
Type of hospital	Primary^a^	3.50±0.52	0.15 (.857)	3.83±0.38	0.49 (.610)	3.67±0.65	0.18 (.833)	3.83±0.38	1.57 (.211)	3.75±0.45	0.56 (.570)	3.92±0.28	3.84 (.024)
	Secondary^b^	3.47±0.78		3.80±0.48		3.63±0.50		3.64±0.50		3.67±0.53		3.66±0.49	(b<c)^‡^
	Tertiary general^c^	3.39±0.49		3.89±0.31		3.57±0.50		3.79±0.41		3.79±0.41		3.89±0.31	
Employment duration in the labor and delivery room (year)	<1^a^	3.44±0.52	2.37 (.073)	3.78±0.44	2.11 (.101)	3.44±0.52	2.52 (.060)	3.67±0.50	1.84 (.142)	3.67±0.50	1.75 (.160)	3.56±0.52	3.31 (.022)
	1–2^b^	3.27±0.91		3.71±0.57		3.50±0.54		3.58±0.53		3.60±0.60		3.62±0.53	(b<c,d)^‡^
	3–4^c^	3.65±0.48		3.92±0.27		3.76±0.43		3.76±0.43		3.84±0.37		3.86±0.34	
	≥5^d^	3.55±0.50		3.90±0.30		3.71±0.52		3.81±0.40		3.74±0.44		3.84±0.37	
Total employment period (year)	<1^a^	3.50±0.57	6.02 (.001)	3.75±0.50	1.70 (.170)	3.25±0.50	1.84 (.143)	3.50±0.57	2.14 (.098)	3.50±0.57	2.21 (.090)	3.50±0.57	3.82 (.012)
	1–2^b^	3.50±0.51	(c<d)^†^	3.71±0.61		3.71±0.46		3.71±0.46		3.64±0.63		3.71±0.61	(c<d)^†^
	3–4^c^	3.13±0.90		3.74±0.29		3.52±0.54		3.57±0.54		3.59±0.58		3.59±0.49	
	≥5^d^	3.68±0.47		3.91±0.29		3.69±0.49		3.78±0.41		3.82±0.39		3.86±0.34	

† Scheffé test,

‡ least significant difference.

**Table 4.** Differences in experience of traumatic perinatal events according to general characteristics (N=129)

Variable	Categories	Dystocia			Postpartum hemorrhage			Neonatal congenital anomalies			Severe maternal or neonatal injury			Stillbirth			Maternal or neonatal death		
		Yes	No	*χ*^2^ (*p*)	Yes	No	*χ*^2^ (*p*)	Yes	No	*χ*^2^ (*p*)	Yes	No	*χ*^2^ (*p*)	Yes	No	*χ*^2^ (*p*)	Yes	No	*χ*^2^ (*p*)
Age (year)	20–29	49 (21.0)	13 (79.0)	0.82 (.662)	55 (88.7)	7 (11.3)	(.095)^†^	38 (61.3)	24 (38.7)	7.76 (.021)	32 (51.6)	30 (48.4)	2.64 (.266)	47 (75.8)	15 (24.1)	5.04 (.080)	27 (43.5)	35 (56.5)	3.02(.221)
	30–39	40 (25.9)	14 (74.1)		53 (98.1)	1 (1.9)		43 (79.6)	11 (20.4)		24 (44.4)	30 (55.6)		38 (70.4)	16 (29.6)		28 (51.9)	26 (48.1)	
	≥40	11 (15.4)	2 (84.6)		13 (100)	0 (0)		12 (92.3)	1 (7.7)		9 (69.2)	4 (30.8)		13 (100)	0 (0)		9 (69.2)	4 (30.8)	
Education level	3-year college	13 (61.9)	8 (38.1)	(.171)^†^	20 (95.2)	1 (4.8)	(.791)^†^	13 (61.9)	8 (38.1)	(.517)^†^	7 (33.3)	14 (66.7)	(.208)^†^	12 (57.1)	9 (42.9)	(.058)^†^	4 (19.0)	17 (81.0)	(.005)^†^
	4-year university	83 (80.6)	20 (19.4)		96 (93.2)	7 (6.8)		76 (73.8)	27 (26.2)		55 (53.4)	48 (46.6)		81 (78.6)	22 (21.4)		57 (55.3)	46 (44.7)	
	Graduate school	4 (80.0)	1 (20.0)		5 (100)	0 (0)		4 (80.0)	1 (20.0)		3 (60.0)	2 (40.0)		5 (100)	0 (0)		3 (60.0)	2 (40.0)	
Midwife license	Yes	29 (82.9)	6 (17.1)	0.78 (.376)	33 (94.3)	2 (5.7)	(1.00)^†^	30 (85.7)	5 (14.3)	4.43 (.035)	18 (51.4)	17 (48.6)	0.02 (.885)	29 (82.9)	6 (17.1)	1.24 (.264)	23 (65.7)	12 (34.3)	4.98 (.026)
	No	71 (75.5)	23 (24.5)		88 (93.6)	6 (6.4)		63 (67.0)	31 (33.0)		47 (50.0)	47 (50.0)		69 (73.4)	25 (26.6)		41 (43.6)	53 (56.4)	
Type of hospital	Primary	8 (66.7)	4 (33.3)	1.17 (.556)	11 (91.7)	1 (8.3)	(.856)^†^	8 (66.7)	4 (33.3)	7.66 (.022)	4 (33.3)	8 (66.7)	3.71 (.156)	7 (58.3)	5 (41.7)	6.83 (.033)	6 (50.0)	6 (50.0)	1.82 (.401)
	Secondary	71 (79.8)	18 (20.2)		83 (93.3)	6 (6.7)		59 (66.3)	30 (33.7)		43 (48.3)	46 (51.7)		65 (73.0)	24 (27.0)		41 (46.1)	48 (53.9)	
	Tertiary general	21 (75.0)	7 (25.0)		27 (96.4)	1 (3.6)		26 (92.9)	2 (7.1)		18 (64.3)	10 (35.7)		26 (92.9)	2 (7.1)		17 (60.7)	11 (39.3)	
Employment duration in the labor and delivery room (year)	<1	4 (44.4)	5 (55.6)	8.77 (.033)	4 (44.4)	5 (55.6)	(<.001)^†^	2 (22.2)	7 (77.8)	22.58 (<.001)	2 (22.2)	7 (77.8)	(.104)^†^	4 (44.4)	5 (55.6)	19.69 (<.001)	0 (0)	9 (100)	(.001)^†^
	1–2	39 (75.0)	13 (25.0)		49 (94.2)	3 (5.8)		32 (61.5)	20 (38.5)		23 (44.2)	29 (55.8)		33 (63.5)	19 (36.5)		21 (40.4)	31 (59.6)	
	3–4	29 (78.4)	8 (21.6)		37 (100)	0 (0)		30 (81.1)	7 (18.9)		20 (54.1)	17 (45.9)		30 (81.1)	7 (18.9)		22 (59.5)	15 (40.5)	
	≥5	28 (90.3)	3 (9.7)		31 (100)	0 (0)		29 (93.5)	2 (6.5)		20 (64.5)	11 (35.5)		31 (100)	0 (0)		21 (67.7)	10 (32.3)	
Total employment period (year)	<1	4 (100)	0 (0)	(.787)^†^	1 (25.0)	3 (75.0)	(<.001)^†^	1 (25.0)	3 (75.0)	(<.001)^†^	1 (25.0)	3 (75.0)	(.293)^†^	2 (50.0)	2 (50.0)	(.006)^†^	0 (0)	4 (100)	(.034)^†^
	1–2	10 (71.4)	4 (28.6)		12 (85.7)	2 (14.3)		5 (35.7)	9 (64.3)		6 (42.9)	8 (57.1)		8 (57.1)	6 (42.9)		4 (28.6)	10 (71.4)	
	3–4	36 (78.3)	10 (21.7)		45 (97.8)	1 (2.2)		31 (67.4)	15 (32.6)		20 (43.5)	26 (56.5)		31 (67.4)	15 (32.6)		22 (47.8)	24 (52.2)	
	≥5	50 (76.9)	15 (23.1)		63 (96.9)	2 (3.1)		56 (86.2)	9 (13.8)		38 (58.5)	27 (41.5)		57 (87.7)	8 (12.3)		38 (58.5)	27 (41.5)	

Values are presented as number (%).

† Fisher exact test.

**Table 5.** Differences in experiences of education on traumatic perinatal events according to general characteristics (N=129)

Variable	Categories	Dystocia			Postpartum hemorrhage			Neonatal congenital anomalies			Severe maternal or neonatal injury			Stillbirth			Maternal or neonatal death		
		Yes	No	*χ*^2^ (*p*)	Yes	No	*χ*^2^ (*p*)	Yes	No	*χ*^2^ (*p*)	Yes	No	*χ*^2^ (*p*)	Yes	No	*χ*^2^ (*p*)	Yes	No	*χ*^2^ (*p*)
Total		115 (89.1)	14 (10.9)		117 (90.7)	12 (9.3)		100 (77.5)	29 (22.5)		103 (78.8)	26 (20.2)		117 (90.7)	12 (9.3)		97 (75.2)	32 (24.8)	
Age (year)	20–29	52 (83.9)	10 (16.1)	4.02 (.133)	54 (87.1)	8 (12.9)	2.51 (.284)	44 (71.0)	18 (29.0)	2.95 (.229)	46 (74.2)	16 (25.8)	2.37 (.306)	54 (87.1)	8 (12.9)	2.51 (.284)	40 (64.5)	22 (35.5)	7.74 (.021)
	30–39	50 (92.6)	4 (7.4)		50 (92.6)	4 (7.4)		45 (83.3)	9 (16.7)		46 (85.2)	8 (14.8)		50 (92.6)	4 (7.4)		45 (83.3)	9 (16.7)	
	≥40	13 (100)	0 (0)		13 (100)	0 (0)		11 (84.6)	2 (15.4)		11 (84.6)	2 (15.4)		13 (100)	0 (0)		12 (92.3)	1 (7.7)	
Education level	3-year college	20 (95.2)	1 (4.8)	(.477)^†^	20 (95.2)	1 (4.8)	(.422)^†^	17 (81.0)	4 (19.0)	(.574)^†^	19 (90.5)	2 (9.5)	(.207)^†^	21 (100)	0 (0)	(.154)^†^	18 (85.7)	3 (14.3)	(.453)^†^
	4-year university	91 (88.3)	12 (11.7)		93 (90.3)	10 (9.7)		80 (77.7)	23 (22.3)		81 (78.6)	22 (21.4)		92 (89.3)	11 (10.7)		75 (72.8)	28 (27.2)	
	Graduate school	4 (80.0)	1 (20.0)		4 (80.0)	1 (20.0)		3 (60.0)	2 (40.0)		3 (60.0)	2 (40.0)		4 (80.0)	1 (20.0)		4 (80.0)	1 (20.0)	
Midwife license	Yes	35 (100)	0 (0)	(.011)^†^	35 (100)	0 (0)	(.036)^†^	33 (94.3)	2 (5.7)	(.004)^†^	33 (94.3)	2 (5.7)	6.22 (.013)	35 (100)	0 (0)	(.036)^†^	32 (91.4)	3 (8.6)	6.78 (.009)
	No	80 (85.1)	14 (14.9)		82 (87.2)	12 (12.8)		67 (71.3)	27 (28.7)		70 (74.5)	24 (25.5)		82 (87.2)	12 (12.8)		65 (69.1)	29 (30.9)	
Type of hospital	Primary	11 (91.7)	1 (8.3)	(1.00)^†^	12 (100)	0 (0)	(.704)^†^	11 (91.7)	1 (8.3)	5.21 (.074)	11 (91.7)	1 (8.3)	1.14 (.563)	12 (100)	0 (0)	(.704)^†^	12 (100)	0 (0)	4.47 (.107)
	Secondary	79 (88.8)	10 (11.2)		79 (88.8)	10 (11.2)		64 (71.9)	25 (28.1)		70 (78.7)	19 (21.3)		79 (88.8)	10 (11.2)		64 (71.9)	25 (28.1)	
	Tertiary general	25 (89.3)	3 (10.7)		26 (92.9)	2 (7.1)		25 (89.3)	3 (10.7)		22 (78.6)	6 (21.4)		26 (92.9)	2 (7.1)		21 (75.0)	7 (25.0)	
Employment duration in the labor and delivery room (year)	<1	5 (55.6)	4 (44.4)	(.009)^†^	6 (66.7)	3 (33.3)	(.083)^†^	3 (33.3)	6 (66.7)	11.14 (.011)	3 (33.3)	6 (66.7)	14.52 (.002)	7 (77.8)	2 (22.2)	(.286)^†^	3 (33.3)	6 (66.7)	11.13 (.011)
	1–2	45 (86.5)	7 (13.5)		47 (90.4)	5 (9.6)		41 (78.8)	11 (21.2)		43 (82.7)	9 (17.3)		47 (90.4)	5 (9.6)		38 (73.1)	14 (26.9)	
	3–4	35 (94.6)	2 (5.4)		34 (91.9)	3 (8.1)		31 (83.8)	6 (16.2)		29 (78.4)	8 (21.6)		33 (89.2)	4 (10.8)		29 (78.4)	8 (21.6)	
	≥5	30 (96.8)	1 (3.2)		30 (96.8)	1 (3.2)		25 (80.6)	6 (19.4)		28 (90.3)	3 (9.7)		30 (96.8)	1 (3.2)		27 (87.1)	4 (12.9)	
Total employment period (year)	<1	3 (75.0)	1 (25.0)	(.221)^†^	3 (75.0)	1 (25.0)	(.171)^†^	1 (25.0)	3 (75.0)	(.130)^†^	1 (25.0)	3 (75.0)	(.082)^†^	4 (100)	0 (0)	(.710)^†^	2 (50.0)	2 (50.0)	(.529)^†^
	1–2	11 (78.6)	3 (21.4)		11 (78.6)	3 (21.4)		11 (78.6)	3 (21.4)		11 (78.6)	3 (21.4)		12 (85.7)	2 (14.3)		10 (71.4)	4 (28.6)	
	3–4	43 (93.5)	3 (6.5)		43 (93.5)	3 (6.5)		36 (78.3)	10 (21.7)		38 (82.6)	8 (17.4)		43 (93.5)	3 (6.5)		34 (73.9)	12 (26.1)	
	≥5	58 (89.2)	7 (10.8)		60 (92.3)	5 (7.7)		52 (80.0)	13 (20.0)		53 (81.5)	12 (18.5)		58 (89.2)	7 (10.8)		51 (78.5)	14 (21.5)	

Values are presented as number (%).

† Fisher exact test.

## References

[1] American Psychiatric Association (2013). Diagnostic and Statistical Manual of Mental Disorders, 5th edition (DSM-5).

[2] Sheen K, Spiby H, Slade P (2016). What are the characteristics of perinatal events perceived to be traumatic by midwives?. Midwifery.

[3] Çankaya S, Dikmen HA (2020). The relationship between posttraumatic stress symptoms of maternity professionals and quality of work life, cognitive status, and traumatic perinatal experiences. Arch Psychiatr Nurs.

[4] Statistic Korea (2022). 2022 Birth statistics [Internet]. https://kosis.kr/statHtml/statHtml.do?orgId=101&tblId=DT_1PO2001&conn_path=I2.

[5] Sheen K, Spiby H, Slade P (2015). Exposure to traumatic perinatal experiences and posttraumatic stress symptoms in midwives: prevalence and association with burnout. Int J Nurs Stud.

[6] Hwang JY (2020). Reclassification of high-risk pregnancy for maternal-fetal healthcare providers. J Korean Matern Child Health.

[7] Aydın R, Aktaş S (2021). Midwives’ experiences of traumatic births: a systematic review and meta-synthesis. Eur J Midwifery.

[8] Lavoie P, Lapierre A, Maheu-Cadotte MA, Rodriguez D, Lavallée A, Mailhot T (2022). Improving the recognition and management of hemorrhage: a scoping review of nursing and midwifery education. Nurse Educ Today.

[9] Utz B, Kana T, van den Broek N (2015). Practical aspects of setting up obstetric skills laboratories--a literature review and proposed model. Midwifery.

[10] Jung GA, Kim MJ (2020). Work performance and calling as factors influencing job satisfaction among nurse midwives working in the delivery room. Korean J Women Health Nurs.

[11] Kim Y, Kim JI, Jeong GH, Kang HS, Kim M, Moon SH (2019). A survey on the educational needs and competence of nurses in maternal fetal intensive care unit. Korean J Women Health Nurs.

[12] Shin S, Park YW, Kim M, Kim J, Lee I (2019). Survey on the education system for new graduate nurses in hospitals: Focusing on the preceptorship. Korean Med Educ Rev.

[13] Jeong S, Hyoung HK (2022). Nursing students’ experience with virtual simulations and clinical practicums for maternity nursing. J Qual Res.

[14] Kim UO, Brousseau DC, Konduri GG (2008). Evaluation and management of the critically ill neonate in the emergency department. Clin Ped Emerg Med.

[15] Lee Y (2017). Work experiences of delivery room nurses: a phenomenological study. Korean J Women Health Nurs.

[16] Ha JH, Kang SJ (2018). Knowledge and educational needs related to an artificial pacemaker among hospital nurses. J Korean Clin Nurs Res.

[17] Kim OS, Jeong SY, Kim JY, So YR (2018). Status of infection control and educational needs of nurses in long term care facilities in Korea. Korean J Rehabil Nurs.

[18] Wahlberg Å, Andreen Sachs M, Johannesson K, Hallberg G, Jonsson M, Skoog Svanberg A (2017). Post-traumatic stress symptoms in Swedish obstetricians and midwives after severe obstetric events: a cross-sectional retrospective survey. BJOG.

[19] Qu L, Gao J, Liu L, Lun B, Chen D (2022). Compassion fatigue and compassion satisfaction among Chinese midwives working in the delivery room: a cross-sectional survey. Midwifery.

[20] Lee JA, Lee SM, Chung SH, Lee JH, Shim JW, Lim JW (2023). Major congenital anomalies in Korean livebirths in 2013-2014: based on the National Health Insurance Database. J Korean Med Sci.

[21] Qian J, Sun S, Wu M, Liu L, Yaping S, Yu X (2021). Preparing nurses and midwives to provide perinatal bereavement care: a systematic scoping review. Nurse Educ Today.

[22] Sheehy A, Baird K (2022). A qualitative study of early career Australian midwives’ encounters with perinatal grief, loss and trauma. Women Birth.

[23] Korean Nursing Association Edu Center (2023). Online supplementary education program [Internet]. https://edu.kna.or.kr/lms/front/courseApply/doDetailCourseView.do?openingclassno=241.

[24] Korean Midwives Association (2023). Korean Midwives Association education center [Internet]. http://www.midwife.or.kr/bbs/board.php?bo_table=education01&wr_id=23.

[25] Kato C, Kataoka Y (2017). Simulation training program for midwives to manage postpartum hemorrhage: a randomized controlled trial. Nurse Educ Today.

[26] Rao KD, Srivastava S, Warren N, Mayra K, Gore A, Das A (2019). Where there is no nurse: an observational study of large-scale mentoring of auxiliary nurses to improve quality of care during childbirth at primary health centres in India. BMJ Open.

[27] Kim M, Ha J (2020). Simulation-based education program on postpartum hemorrhage for nursing students. Korean J Women Health Nurs.

[28] Shim CS, Park SY (2018). Effects of a simulator-based delivery education on the major satisfaction, nursing professionalism and clinical competence of the nursing students. J Korea Entertain Ind Assoc.

[29] Park KY (2020). Trend analysis of studies on simulation-based education for delivery nursing: based on Jeffries’s simulation model. J Soc Converg Stud.

[30] Korean Nursing Association Edu Center (2023). Offline supplementary education program [Internet]. https://edu.kna.or.kr/lms/front/programApply/doListProgramApplyDetailView.do?mnid=201609595175&prgmno=26311&prgmexecuteno=32734.

